# Soluble histone H2AX is induced by DNA replication stress and sensitizes cells to undergo apoptosis

**DOI:** 10.1186/1476-4598-7-61

**Published:** 2008-07-10

**Authors:** Ying Liu, Joshua A Parry, Anna Chin, Stefan Duensing, Anette Duensing

**Affiliations:** 1 Molecular Virology Program, University of Pittsburgh Cancer Institute, Pittsburgh, PA, 15213, USA; 2 Department of Microbiology and Molecular Genetics, University of Pittsburgh School of Medicine, Pittsburgh, PA, 15261, USA; 3 Department of Pathology, University of Pittsburgh School of Medicine, Pittsburgh, PA, 15261, USA; 4 University of Pittsburgh Cancer Institute, Hillman Cancer Center, Research Pavilion Suite 1.8, 5117 Centre Avenue, Pittsburgh, PA, 15213, USA

## Abstract

**Background:**

Chromatin-associated histone H2AX is a key regulator of the cellular responses to DNA damage. However, non-nucleosomal functions of histone H2AX are poorly characterized. We have recently shown that soluble H2AX can trigger apoptosis but the mechanisms leading to non-chromatin-associated H2AX are unclear. Here, we tested whether stalling of DNA replication, a common event in cancer cells and the underlying mechanism of various chemotherapeutic agents, can trigger increased soluble H2AX.

**Results:**

Transient overexpression of H2AX was found to lead to a detectable fraction of soluble H2AX and was associated with increased apoptosis. This effect was enhanced by the induction of DNA replication stress using the DNA polymerase α inhibitor aphidicolin. Cells manipulated to stably express H2AX did not contain soluble H2AX, however, short-term treatment with aphidicolin (1 h) resulted in detectable amounts of H2AX in the soluble nuclear fraction and enhanced apoptosis. Similarly, soluble endogenous H2AX was detected under these conditions. We found that excessive soluble H2AX causes chromatin aggregation and inhibition of ongoing gene transcription as evidenced by the redistribution and/or loss of active RNA polymerase II as well as the transcriptional co-activators CBP and p300.

**Conclusion:**

Taken together, these results show that DNA replication stress rapidly leads to increased soluble H2AX and that non-chromatin-associated H2AX can sensitize cells to undergo apoptosis. Our findings encourage further studies to explore H2AX and the cellular pathways that control its expression as anti-cancer drug targets.

## Background

Chromatin-associated histone H2AX is a key regulator of the cellular response to genotoxic stress and DNA double-strand breaks [1-6]. H2AX is a core histone H2A variant that is randomly incorporated into nucleosomes during DNA replication. It differs from histone H2A by a unique C-terminal tail that contains a highly conserved SQE motif with a serine residue at position 139 [7]. Serine 139 becomes rapidly phosphorylated in response to DNA double strand breaks by protein kinases of the phosphatidylinositol 3-OH-kinase-related kinase (PI3KK) family including ATM, ATR or DNA-PK [8-10]. The phosphorylated form of H2AX has been referred to as γ-H2AX [2]. Several lines of evidence suggest that the main function of histone H2AX is to orchestrate the DNA damage response through interactions with BRCT repeat-containing proteins that bind phosphorylated H2AX such as MDC1 [11-13]. However, H2AX has also been implicated in a number of other functions including inactivation of sex chromosomes and control of sister chromatid recombination [14,15]. We have recently shown that histone H2AX also has non-nucleosomal functions, specifically, pro-apoptotic activities in gastrointestinal stromal tumor (GIST) cells treated with the small molecule protein kinase inhibitor imatinib mesylate (Gleevec) [16].

Previous studies in yeast have shown that histone levels need to be tightly regulated and that high levels of non-chromatin bound histones are cytotoxic [17]. Soluble histones can accumulate when DNA replication and histone incorporation into the newly synthesized chromatin become uncoupled, for example after stalling of replication forks [17-19].

The present study was designed to determine the mechanisms that lead to increased soluble H2AX. We found that even short-term stalling of replication forks is sufficient to cause an increase of soluble H2AX. Furthermore, we show that transient overexpression of H2AX, but not H2A, promotes apoptotic cell death. In addition, we demonstrate that overexpression of H2AX leads to a nuclear redistribution and/or loss of the active form of RNA polymerase II as well as the transcriptional co-activators CBP and p300. Taken together, our results suggest a model in which replication stress leads to increased soluble H2AX, which sensitize cells to undergo apoptosis in a process that involves a shut-down of gene transcription.

## Results

### Replication stress induces soluble H2AX

**Figure 1:**
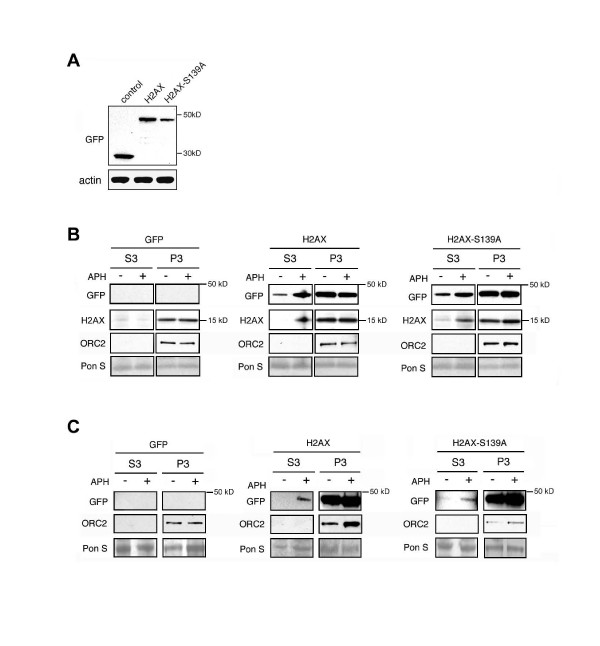
**DNA replication stress induces soluble H2AX**. (A) Immunoblot analysis for GFP of U-2 OS cells stably transfected with empty GFP vector (control), GFP-H2AX or GFP-H2AX-S139A for 24 h. (B,C) Immunoblot analysis of soluble nuclear (S3) and chromatin bound (P3) protein fractions obtained from U-2 OS cells transiently (B; 24 h) or stably (C) transfected with empty GFP vector, GFP-H2AX or GFP-H2AX-S139A and treated with 10 μM aphidicolin (APH) for 1 h to block DNA replication. Staining for GFP detects expression of tagged proteins, whereas staining for H2AX detects endogenous protein. Immunoblot for ORC2 is shown to rule out carry-over between fractions. Ponceau S staining demonstrates protein loading.

To test whether DNA replication stress can induce soluble H2AX, we used the human osteosarcoma cell line U-2 OS that, unlike GIST cells, does not depend on a single oncogenically activated tyrosine kinase such as KIT and has *bona fide *normal DNA damage response pathways. Cells were transfected with constructs encoding GFP-tagged H2AX, mutant GFP-H2AX-S139A or empty GFP vector [20]. Expression of overexpressed H2AX proteins was distinguishable from endogenous H2AX based on the increased molecular weight (Fig. 1A). After transient transfection (24 h), a considerable amount of GFP-tagged H2AX proteins were detected in the soluble nuclear compartment (Fig. 1B; lanes S3, untreated, middle and right panels). When these cells were treated with aphidicolin, a DNA polymerase α inhibitor that induces stalling of replication forks, a rapid increase of soluble proteins was detected in the S3 fractions within 1 h (Fig. 1B). The ribonucleotide reductase inhibitor hydroxyurea (HU), which induces replication stress through nucleotide depletion, had a similar effect (data not shown). Treatment with aphidicolin or HU also led to an increase of endogenous H2AX in the soluble fractions (Fig. 1B). These results highlight previous findings in yeast showing excess levels of free histones in cells in which DNA replication and histone deposition are uncoupled [17-19]. Transfection with mutant H2AX in which the phosphorylation site at S139 has been abolished (H2AX-S139A) had a similar effect as wild-type H2AX, suggesting that the increase of soluble H2AX is not dependent on this phosphorylation site. To test whether our results allow generalization, we transiently transfected the human lung cancer cell line H1299 with GFP-tagged H2AX, H2AX-S139A and empty vector. Treatment with aphidicolin for 1 h, also led to an increased fraction of soluble H2AX indicating that his response is not cell type specific (Additional File 1).

To further corroborate our results, U-2 OS cell populations stably expressing GFP-tagged H2AX, H2AX-S139A or empty vector were generated. In stable cell populations, H2AX was undetectable in S3 fractions from untreated cells (Fig. 1C). However, treatment with aphidicolin for 1 h rapidly resulted in an increase of soluble H2AX and H2AX-S139A (Fig. 1C; S3 fractions).

Taken together, these results show that transient stalling of DNA replication forks is associated with an increase of soluble histone H2AX.

### Overexpression of H2AX sensitizes cells to undergo apoptosis

**Figure 2:**
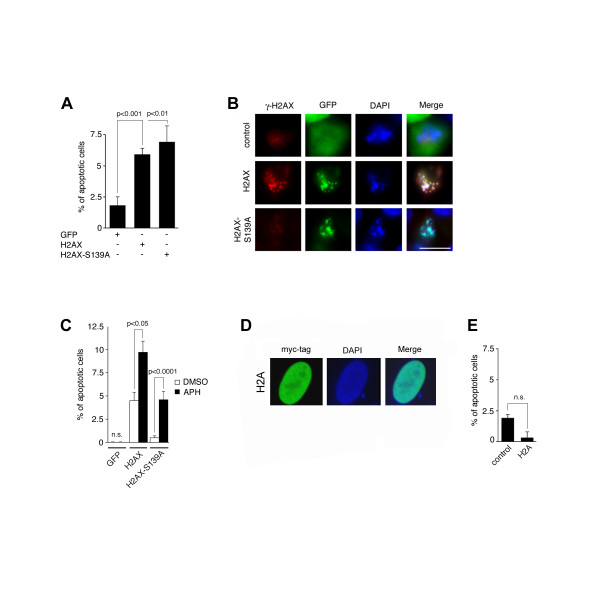
**Overexpression of H2AX but not H2A leads to apoptotic cell death and stimulates apoptosis**. (A) Quantification of apoptotic U-2 OS cells following transient overexpression of empty GFP vector, GFP-H2AX or GFP-H2AX-SA for 24 h. Only transfected, GFP-positive cells were counted. Apoptosis was scored based on the nuclear morphology. Each bar indicates mean + standard error of at least three independent experiments with at least 100 cells counted per experiment. (B) Fluorescence microscopic analysis of U-2 OS cells transiently transfected with empty GFP vector (control), GFP-H2AX or GFP-H2AX-S139A for expression of γ-H2AX. Note the negative staining for γ-H2AX of a cell transfected with mutant H2AX-S139A. Nuclei stained with DAPI. Scale bar indicates 10 μm. (C) Quantification of apoptotic cells in U-2 OS populations stably transfected with empty GFP vector, GFP-H2AX or GFP-H2AX-S139A and treated with 10 μM aphidicolin for 24 h. Each bar indicates mean + standard error of at least three independent experiments with at least 100 cells counted per experiment. (D) Fluorescence microscopic analysis of a U-2 OS cell transiently transfected with myc-H2A and stained with an anti-myc-tag antibody. Nucleus stained with DAPI. Scale bar indicates 10 μm. (E) Quantification of apoptotic U-2 OS cells following transient overexpression of empty myc-tag vector (control) or myc-H2A for 24 h. Each bar indicates mean + standard error of at least three independent experiments with at least 100 cells counted per experiment.

We next tested whether an increase of soluble H2AX can lead to enhanced apoptosis. As shown in Fig. 1B, transient overexpression (24 h) of H2AX is associated with a proportion of H2AX remaining in the soluble nuclear fraction. At the same time, we detected a significant 3.3-fold increase of apoptotic cells (5.9%; p ≤ 0.001) in comparison to empty vector controls (1.8%; Fig. 2A,B). Apoptotic cells were identified based on the characteristic nuclear morphology including DNA condensation and fragmentation and only GFP-positive, transfected cells were counted (Fig. 2B). Likewise, overexpression of mutant H2AX-S139A also caused a 3.8-fold increase of apoptotic cells (6.9%; p ≤ 0.01; Fig. 2A,B). Hence, apoptosis induction does not critically depend on serine 139 phosphorylation, which is underscored by the finding that cells transfected with H2AX-S139A lacked immunostaining for γ-H2AX but still underwent apoptosis (Fig. 2B; bottom panels).

To determine whether replication stress sensitizes H2AX-overexpressing cells to undergo apoptosis, we used U-2 OS cell populations stably expressing either empty GFP vector, GFP-tagged H2AX or GFP-H2AX-S139A. Cells were treated with aphidicolin for 24 h and the proportion of apoptotic cells was assessed (Fig. 2C). A significant 2.2-fold increase of apoptotic U-2 OS cells stably expressing GFP-H2AX was detected from 4.5% in DMSO-treated controls to 9.7% in aphidicolin-treated populations (p ≤ 0.05). In addition, a significant 9.2-fold increase of apoptotic cells was detected in U-2 OS cells stably expressing GFP-H2AX-S139A from 0.5% in DMSO-treated controls to 4.6% in aphidicolin-treated cells (p ≤ 0.0001). No apoptotic cells were found in populations expressing empty GFP vector, regardless of the drug treatment (Fig. 2C).

We tested next whether overexpression of core histone H2A was able to stimulate apoptosis. Transiently transfected cell populations were stained with an anti-myc-tag antibody and assessed for apoptotic nuclear morphology. The proportion of transfected cells was similar to H2AX-transfected populations. No enhanced cell death was detected when myc-tagged H2A was overexpressed under transient conditions in comparison to empty myc-tag vector (Fig. 2D,E).

Taken together, these results indicate that overexpression of histone H2AX, but not histone H2A, can sensitize cells to undergo apoptosis.

### Overexpression of H2AX causes chromatin aggregation

**Figure 3:**
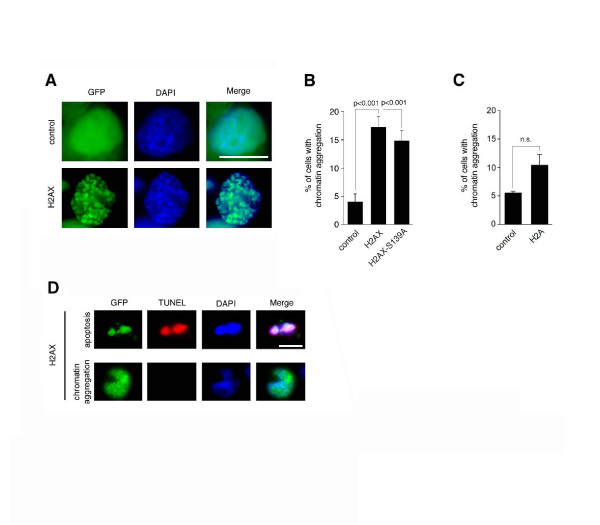
**Overexpression of H2AX leads to aberrant chromatin aggregation and apoptosis**. (A) Fluorescence microscopic analysis of U-2 OS cells transiently overexpressing empty GFP vector (control) or GFP-H2AX for 24 h. Note the chromatin aggregation in cells transfected with H2AX. Nuclei stained with DAPI. Scale bar indicates 10 μm. (B,C) Quantification of U-2 OS cells with chromosome aggregation 24 h after transfection with empty GFP vector (control in B), GFP-H2AX, GFP-H2AX-S139A, empty myc-tag vector (control in C) or myc-H2A. Each bar indicates mean + standard error of at least three independent experiments with at least 100 cells counted per experiment. (D) TUNEL staining of U-2 OS cells transiently transfected with GFP-H2AX and either showing an apoptotic morphology (top) or chromatin aggregation (bottom). Note that the cell with chromatin aggregation is TUNEL-negative. Nuclei stained with DAPI. Scale bar indicates 10 μm.

In order to determine the mechanisms that underlie H2AX induced cell death, we performed a morphological analysis of U-2 OS cells transiently transfected with empty GFP vector, GFP-H2AX or GFP-H2AX-S139A (Fig. 3A). Excessive amounts of histones have previously been shown to result in non-specific binding to DNA and/or chromatin leading to structural alterations [16,21]. In transient transfection experiments, we detected an increase of cells with aberrant chromatin aggregation from 4% in empty vector controls to 17.2% in H2AX-transfected cells (4.3-fold; p ≤ 0.001) and 14.8% H2AX-S139A-transfected cells (3.7-fold; p ≤ 0.001; Fig. 3A,B). U-2 OS cells transiently transfected with core histone H2A showed a moderate increase of chromatin aggregation from 5.5% in empty vector controls to 10.4% in H2A-transfected cells that was not statistically significant (Fig. 3C). Chromatin aggregation induced by H2AX was not equivalent to apoptosis since cells with chromatin aggregation did not show TUNEL positivity as exemplified in Fig. 3D (bottom panels).

### Overexpression of histone H2AX is associated with a redistribution of components of the transcriptional machinery

**Figure 4:**
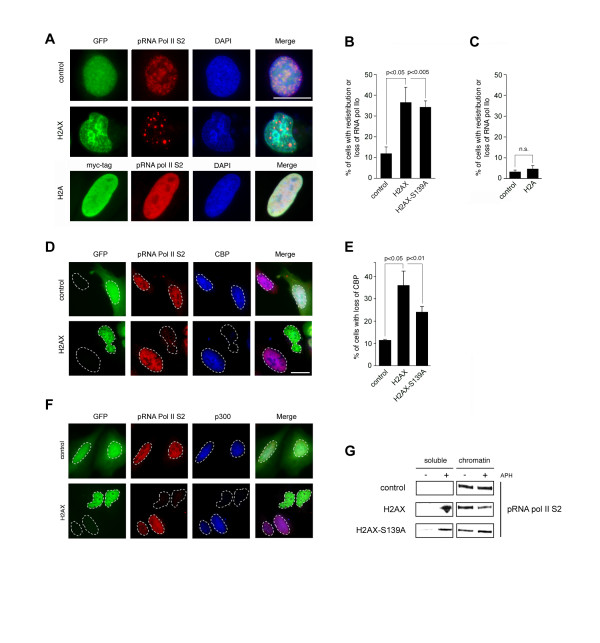
**H2AX leads to a redistribution of the transcriptional machinery**. (A) Immunofluorescence analysis of U-2 OS cells for expression of pRNA pol II S2 (Covance, Berkeley, CA) 24 h after transfection with empty GFP vector (control), GFP-H2AX or myc-H2A. Note chromatin aggregation and the speckled pRNA Pol II S2 pattern in cells transfected with H2AX. Nuclei stained with DAPI. Scale bar indicates 10 μm. (B,C) Quantification of U-2 OS cells with redistributed or lost RNA pol II S2 expression 24 h after transfection with empty GFP vector (control in B), GFP-H2AX, GFP-H2AX-S139A, empty myc-tag vector (control in C) or myc-H2A. Each bar indicates mean + standard error of at least three independent experiments with at least 100 cells counted per experiment. (D) Double-immunofluorescence analysis of U-2 OS cells for expression of pRNA pol II S2 and CBP (Santa Cruz, Santa Cruz, CA) 24 h after transfection with empty GFP vector (control) or GFP-H2AX. Note the severely reduced expression of pRNA pol II S2 and CBP in the H2AX-transfected cell. Scale bar indicates 10 μm. (E) Quantification of U-2 OS cells with reduced CBP expression 24 h after transfection with empty GFP vector (control), GFP-H2AX, or GFP-H2AX-S139A. Each bar indicates mean + standard error of at least three independent experiments with at least 100 cells counted per experiment. (F) Double-immunofluorescence analysis of U-2 OS cells for expression of pRNA pol II S2 and p300 24 h after transfection with empty GFP vector (control) or GFP-H2AX. Note the severely reduced expression of pRNA pol II and p300 in H2AX-expressing cells. Scale bar indicates 10 μm. (G) Immunoblot analysis of pRNA pol II S2 in soluble nuclear (S3) and chromatin bound (P3) protein fractions obtained from U-2 OS cells transiently transfected (24 h) with empty GFP vector, GFP-H2AX or GFP-H2AX-S139A and treated with 10 μM aphidicolin (APH) for 1 h to block DNA replication.

We lastly asked whether chromatin aggregation induced by overexpression of histone H2AX can lead to a downregulation of active gene transcription [16]. The majority of cellular mRNA transcription is carried out by RNA pol II. The C-terminal domain (CTD) of RNA pol II consists of tandem heptad repeats with the consensus sequence Y_1_S_2_P_3_T_4_S_5_P_6_S_7 _that become hyperphosphorylated and hypophosphorylated during the transcription cycle. Phosphorylation at serine 5 is associated with transcription initiation whereas serine 2 phosphorylation is predominantly found during transcription elongation [22]. Active gene transcription can be visualized by immunofluorescence microscopy against RNA pol II S2 and typically results in a fine granular nuclear staining pattern (Fig. 4A; top panels) [23,24]. The transcriptional co-activators CREB-binding protein (CBP) and p300 regulate RNA pol II-mediated gene transcription by connecting sequence-specific transcription factors to the basal transcription machinery [25]. These proteins display a similar staining pattern during ongoing transcription as RNA pol II. Transcriptional inhibition has been shown to induce a redistribution of the S2 phosphorylated elongating form of RNA pol II (pRNA pol II S2) into speckled nuclear domains and to lead to RNA pol II degradation [26,27]. Likewise, CBP and p300 are redistributed into nuclear speckles upon transcriptional inhibition [27].

We detected a significant increase of cells with redistribution of pRNA pol II S2 into nuclear speckles or loss of pRNA pol II S2 expression below detection level by immunofluorescence microscopy in cells ectopically expressing H2AX (2.9-fold; 36.5%; p ≤ 0.05) or H2AX-S139A (2.7-fold; 34.2%; p ≤ 0.005) when compared to controls (12.5%; Fig. 4A,B). In the vast majority of these cells, nuclear redistribution of RNA pol II S2 coincided with chromatin aggregation (Fig. 4A; middle panels). No significant effect on pRNA pol II S2 distribution was detected when H2A-transfected cells were analyzed (Fig. 4A,B). In addition to pRNA pol II S2, we found the transcriptional co-activator CBP to be displaced from chromatin in cells transfected either with H2AX (3.2-fold increase, 36%; p ≤ 0.05) or H2AX-S139A (2.1-fold increase, 24%; p ≤ 0.01) in comparison to empty vector controls (11.4%; Fig. 4D,E). Similar results were found when cells were analyzed for expression of p300 (Fig. 4F). Furthermore, pRNA pol II S2 was displaced from chromatin after treatment with aphidicolin as shown by immunoblotting further corroborating the results obtained by immunofluorescence staining (Fig. 4G). Since transcription is required for cell viability, our results suggest that the ability of H2AX to abrogate ongoing transcription may sensitize cells to undergo apoptosis.

## Discussion

Histone variant H2AX plays a pivotal role in the maintenance of genome stability, in particular in the absence of p53 [1,6,28]. We have previously shown that soluble histone H2AX is involved in apoptosis induction in GIST cells treated with the small molecule inhibitor imatinib mesylate [16]. Here, we extend these results by showing DNA replication stress as one mechanism that can lead to an increase of the pool of soluble H2AX. We show that overexpression of H2AX sensitizes cells to apoptosis in a process that is associated with a disruption of ongoing gene transcription as evidenced by a redistribution of active RNA pol II as well as CBP and p300.

Our report raises a number of questions that warrant further investigation. First, it is unclear why the overall level of apoptosis in U-2 OS after transient overexpression of H2AX is comparatively low (in the range of 5%) in comparison to GIST cells (approximately 40%) [16]. One possibility is that GIST cells are particularly sensitive to increased non-chromatin associated H2AX because they express oncogenic KIT. We have previously speculated that KIT may constitutively downregulate H2AX, which may render GIST cells particularly sensitive to its re-expression (see also below). Second, despite our current results the precise mechanisms that lead to soluble H2AX remain unclear. Since the reagents used here induce stalling of DNA replication forks, it is possible that the uncoupling of histone production and histone incorporation into chromatin leads to excessive levels of free histones as it has previously been suggested [17-19]. However, prolonged replication arrest can also cause DNA breakage and hence the possibility that increased soluble H2AX is a result of accelerated histone eviction during DNA damage-associated chromatin reorganization cannot be dismissed [29]. Third, the precise mechanisms of apoptosis induction by histone H2AX await further clarification. Our results suggest that chromatin aggregation and transcriptional shut down, which cannot be sustained for a prolonged period of time without affecting cell viability, are important. However, this is a relatively non-specific mechanism and other, more direct, pro-apoptotic mechanisms of H2AX may also be involved [30]. Although we cannot rule out the possibility that release of histones from cells undergoing apoptosis contributes to a certain degree to the increase of soluble H2AX [31], the present report provides additional evidence that soluble histone H2AX is a cause for increased cell death and not simply a consequence.

It is conceivable that replication stress is a frequent event in tumor cells [32,33]. Given the potentially deleterious consequences of excessive H2AX [16,17], one prediction would be that mechanisms exist that regulate the pool of non-chromatin-associated H2AX. Such mechanisms are likely to involve the ubiquitin/proteasome machinery since we have previously shown that H2AX can become polyubiquitinated and that proteasome inhibition can stabilize H2AX [16]. Recent findings show that the ubiquitin-conjugating enzyme Ubc13 and the RING-finger ubiquitin ligase RNF8 are involved in DNA damage-induced H2AX ubiquitination [34-38]. Whether these proteins are also involved in the modulation of H2AX levels in tumor cells remains to be tested.

The fact that soluble H2AX induces apoptosis in tumor cells opens new avenues for cancer therapy. Identification of drugs that induce soluble H2AX, e.g. by inhibiting a potential suppressor of H2AX, would be promising. Given the role of the ubiquitin-proteasome system in the regulation of H2AX levels in GISTs [16], it is also conceivable that targeting the protein degradation machinery could lead to increased soluble H2AX levels. Furthermore, the GFP-tagged H2AX vectors could be used in a compound screen with chromatin aggregation as the readout.

In summary, we report here that DNA replication stress rapidly leads to an increase of soluble H2AX. Overexpression of H2AX was found to sensitize cells to undergo apoptosis, which very likely involves the disruption of ongoing gene transcription. Our findings encourage further studies to exploit H2AX and the cellular mechanisms that regulate its expression levels as novel anti-cancer drug targets.

## Conclusion

This report shows that stalling of DNA replication leads to an increase of soluble H2AX in human tumor cells. Soluble H2AX was found to sensitize cells to undergo apoptosis and this activity was associated with a transcriptional shut-down. These results encourage further studies to exploit histone H2AX and the mechanisms that regulate its expression as anti-cancer drug targets.

## Materials and methods

### Cell culture and transfections

U-2 OS osteosarcoma cells and H1299 lung cancer cells were obtained from ATCC (Manassas, VA) and maintained in Dulbecco's modified Eagle medium (DMEM; Cambrex, Baltimore, MD) supplemented with 10% fetal bovine serum (FBS; Mediatech, Herndon, VA), 50 U/ml penicillin and 50 μg/ml streptomycin (both BioWhittaker, Baltimore, MD). Cells were transfected with an empty GFP vector or N-terminal GFP fusion plasmids encoding wild-type H2AX and H2AX in which serine 139 has been substituted by alanine (H2AX-S139A)[20] using Fugene 6 (Invitrogen, Carlsbad, CA). In addition, empty myc-tag vector or myc-H2A was used for transient overexpression experiments. Stable cell populations were generated by selecting cells in G418-containing media for 48 h.

### Immunological Methods

Whole cell lysates were prepared by scraping cells into RIPA buffer (1% NP-40, 50 mM Tris-HCl pH 8.0, 100 mM sodium fluoride, 30 mM sodium pyrophosphate, 2 mM sodium molybdate, 5 mM EDTA, 2 mM sodium orthovanadate) containing protease inhibitors (10 μg/ml aprotinin, 10 μg/ml leupeptin, 1 μM phenylmethylsulfonyl fluoride). Suspensions were incubated for one hour at 4°C and cleared by centrifugation for 30 min at 14,000 rpm at 4°C. Protein concentrations were determined by the Bradford assay (Biorad, Hercules, CA). 30 μg of protein were loaded on a 4–12% Bis-Tris gel (Invitrogen, Carlsbad, CA) and blotted onto a nitrocellulose membrane. Primary antibodies used were directed against GFP (Lab Vision, Fremont, CA), H2AX (Bethyl Laboratories, Montgomery, TX), ORC2 (BD Biosciences Pharmingen), phosphorylated RNA pol II S2 (Covance, Berkeley, CA) and actin (Sigma, St. Louis, MS).

For immunofluorescence micoscopic analyses, cells were grown on coverslips, washed in PBS, fixed in 4% paraformaldehyde/PBS (15 min), permeabilized with 1% Triton-X 100/PBS (15 min) and blocked in 10% normal donkey serum (Jackson Immunoresearch, West Grove, PA) for 15 min at room temperature. After incubation with primary antibodies overnight at 4°C in a humid chamber, cells were incubated with FITC-conjugated or Rhodamine Red-conjugated secondary antibodies (Jackson Immunoresearch, 2 hours at 37°C) and counterstained with DAPI (Vector Laboratories, Burlingame, CA). For double-immunofluorescence analysis, cells were stained for the first antigen followed by a second primary antibody and an AMCA-labeled secondary antibody (Jackson Immunoresearch). Primary antibodies used for immunofluorescence stainings were directed against γ-H2AX (Trevigen, Gaithersburg, MD), myc-tag (Cell Signaling, Danvers, MA), phosphorylated RNA pol II S2 (Covance, Berkeley, CA), CBP and p300 (both Santa Cruz, Santa Cruz, CA). Cells were analyzed using an Olympus AX70 epifluorescence microscope equipped with a SpotRT digital camera.

### Subcellular fractionation

Subcellular fractionation was performed according to Mendez and Stillman [39]. Cells were trypsinized, collected by centrifugation, washed with PBS, resuspended in Buffer A (10 mM HEPES, pH 7.9, 10 mM KCl, 1.5 mM MgCl_2_, 10 mM NaF, 340 mM sucrose, 10% glycerol, 1 mM DTT, 0.1 mM PMSF, 1 mM sodium orthovanadate, 0.1% Triton-X 100, 5 μg/ml leupeptin 5 μg/ml aprotinin) and incubated on ice for 5 minutes. The nuclei (P1) were collected by low speed centrifugation at 1,300 g and lysed in Buffer B (3 mM EDTA, 0.2 mM EGTA, 1 mM DTT, 0.1 mM PMSF, 1 mM sodium orthovanadate, 5 μg/ml leupeptin, 5 μg/ml aprotinin) for 10 minutes on ice. The insoluble chromatin fraction (P3) was collected by centrifugation at 1,700 g. The supernatant contained the soluble nuclear proteins (S3). The chromatin pellet (P3) was resuspended in SDS sample buffer and the DNA was sheared by sonication in a 550 Sonic Dismembrator (Fisher) at 15% amplitude.

### TUNEL staining

Apoptotic cells were visualized using the In Situ Cell Death Detection Kit (Roche Applied Sciences, Indianapolis, IN) according to manufacturer's instructions.

### Statistical analysis

Statistical significance was assessed using Student's t test for independent samples. P values ≤ 0.05 were considered significant.

## Competing interests

The authors declare that they have no competing interests.

## Authors' contributions

YL, JAP and AC carried out the immunoblot and immunofluorescence experiments and participated in the interpretation of results. SD analyzed the immunofluorescence experiments and participated in the design and interpretation of experiments. AD designed this study, participated in the analysis of experiments and drafted the manuscript.

## Supplementary Material

Additional File 1 DNA replication stress induces soluble H2AX in H1299 lung carcinoma cells. Immunoblot analysis of soluble nuclear (S3) and chromatin bound (P3) protein fractions obtained from H1299 cells transiently transfected with empty GFP vector, GFP-H2AX or GFP-H2AX-S139A and treated with 10 μM aphidicolin (APH) for 1 h to block DNA replication. Staining for GFP detects expression of tagged proteins, whereas staining for H2AX detects endogenous protein. Immunoblot for ORC2 is shown to rule out carry-over between fractions. Ponceau S staining demonstrates protein loading. Click here for file
