# Supplementary figures and images for: Soluble histone H2AX is induced by DNA replication stress and sensitizes cells to undergo apoptosis
Source: Mol Cancer. 2008 Jul 10;7:61. doi: 10.1186/1476-4598-7-61 (PMC2481267; doi:10.1186/1476-4598-7-61)

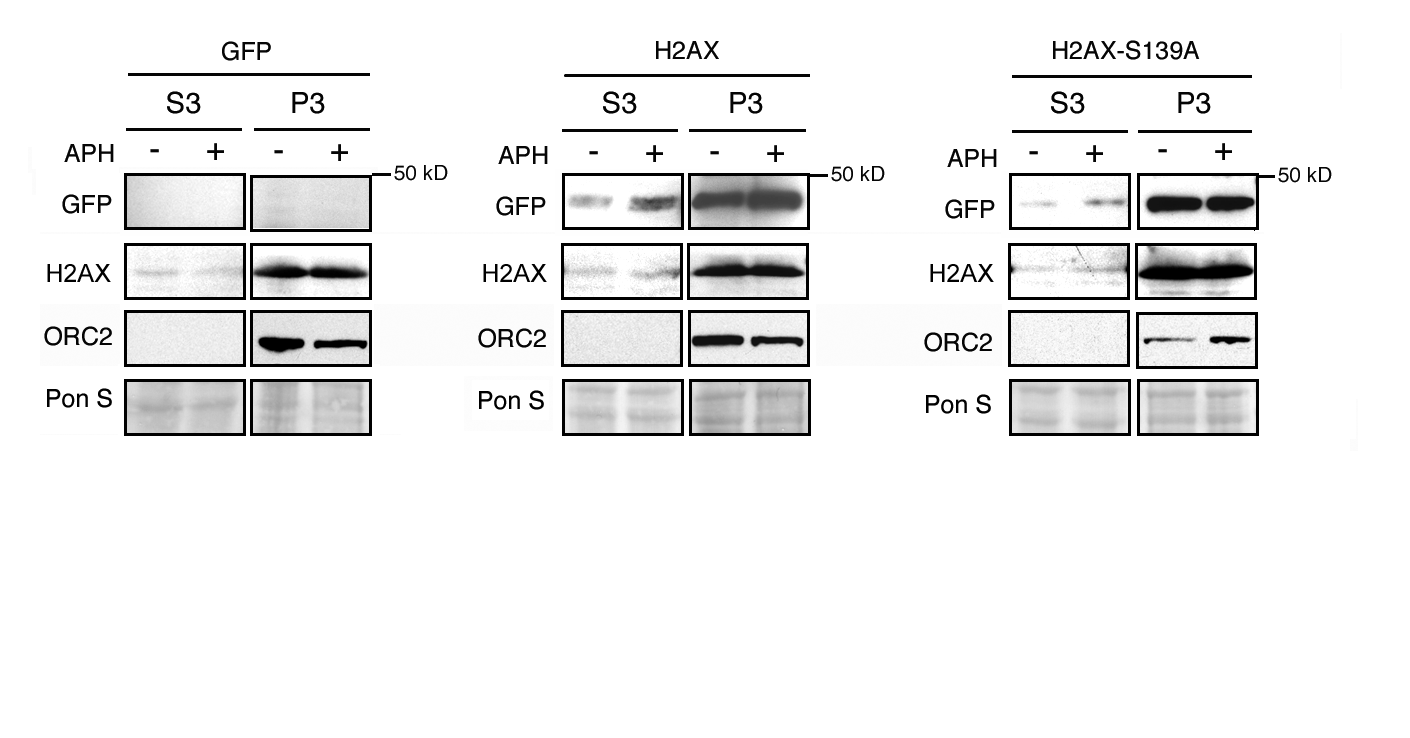

Supplement: Additional File 1 — DNA replication stress induces soluble H2AX in H1299 lung carcinoma cells. Immunoblot analysis of soluble nuclear (S3) and chromatin bound (P3) protein fractions obtained from H1299 cells transiently transfected with empty GFP vector, GFP-H2AX or GFP-H2AX-S139A and treated with 10 μM aphidicolin (APH) for 1 h to block DNA replication. Staining for GFP detects expression of tagged proteins, whereas staining for H2AX detects endogenous protein. Immunoblot for ORC2 is shown to rule out carry-over between fractions. Ponceau S staining demonstrates protein loading. [file 1476-4598-7-61-S1.tiff]
